# Supplementary material for: Longitudinal serologic and viral testing post–SARS-CoV-2 infection and post-receipt of mRNA COVID-19 vaccine in a nursing home cohort—Georgia, October 2020‒April 2021
Source: PLoS One. 2022 Oct 27;17(10):e0275718. doi: 10.1371/journal.pone.0275718 (PMC9612440; doi:10.1371/journal.pone.0275718)
Supplement: S1 Table — (DOCX) [file pone.0275718.s001.docx]

**S1 Table.** **Median** **anti**–**SARS-CoV-2 serum antibody titers and ratios post-vaccination and post-infection by isotype among participants (N=11) in a nursing home cohort—Georgia, October 2020‒April 2021^*^**

| **Time period (days)** | **Post-infection** | | **Post-vaccination** | | **Ratio post-vaccination to post-infection** |
| --- | --- | --- | --- | --- | --- |
|  | **No. specimens tested** | **Median titer (IQR)** ^†^ | **No. specimens tested** | **Median titer**  **(IQR)**^‡§^ |  |
| *Pan-Ig* |  |  |  |  |  |
| 0–14 | 5 | 286 (257–1803) | 9 | 5137 (3584–73070) | 18.0 |
| 15–30 | 7 | 1,441 (1289–3682) | 8 | 299,721 (240573–345136) | 208.0 |
| 31–60 | 9 | 4,769 (2832–7582) | 9 | 64,344 (55171–127753) | 13.5 |
| 61–90 | 3 | 4,763 (2832–6153) | 4 | 53,664 (38454–71967) | 11.3 |
|  |  |  |  |  |  |
| *IgG* |  |  |  |  |  |
| 0–14 | 5 | 395 (298–3340) | 9 | 11,758 (5797–25900) | 29.8 |
| 15–30 | 7 | 3,563 (2662–8028) | 8 | 206,062 (95411–1258378) | 57.8 |
| 31–60 | 9 | 7,472 (4952–15971) | 9 | 380,248 (318681–8705015) | 50.9 |
| 61–90 | 3 | 6,973 (6495–7217) | 4 | 130,964 (73799–185779) | 18.8 |
|  |  |  |  |  |  |
| *IgA* |  |  |  |  |  |
| 0–14 | 3 | 155 (134–223) | 3 | 399 (288–645) | 2.6 |
| 15–30 | 7 | 318 (277–697) | 8 | 2,777 (1178–4462) | 8.7 |
| 31–60 | 7 | 198 (154–1255) | 9 | 1,956 (754–4242) | 9.9 |
| 61–90 | 3 | 399 (379–445) | 4 | 656 (272–1742) | 1.6 |

Abbreviations: IQR = interquartile range

^*^ The post-infection period was defined as the time from SARS-CoV-2 diagnosis (by BinaxNOW™ COVID-19 Ag Cards and/or real-time reverse transcription polymerase chain reaction) to receipt of first Pfizer-BioNTech COVID-19 Vaccine dose. The post-vaccination period was defined as the time after receipt of first vaccine dose to the end of the evaluation period. Individuals with serum antibody titers below the seroconversion threshold (defined as a signal threshold >1 at the 1:100 dilution for any isotype) were not included in median and ratio calculations.

^†^ Median post-infection titers peaked during 15–30 days for IgM, 31–60 days for Pan-Ig and IgG, and 61–90 days for IgA.

^‡^ Participant C was not included in the post-vaccination analyses due to vaccination refusal. This participant’s titers ranged from 2437–16977 for Pan-Ig, 4919–7045 for IgG, and 267–710 for IgA during the 90–150 days post-infection; their IgM titers were below the limit of detection during this time period.

^§^ Median post-vaccination titers peaked during 15–30 days for Pan-Ig and IgA, and 31–60 days for IgG. Post-vaccination median IgM titers were not calculated because most post-vaccination titers were below the limit of detection.
