# Supplementary figures and images for: Longitudinal serologic and viral testing post–SARS-CoV-2 infection and post-receipt of mRNA COVID-19 vaccine in a nursing home cohort—Georgia, October 2020‒April 2021
Source: PLoS One. 2022 Oct 27;17(10):e0275718. doi: 10.1371/journal.pone.0275718 (PMC9612440; doi:10.1371/journal.pone.0275718)

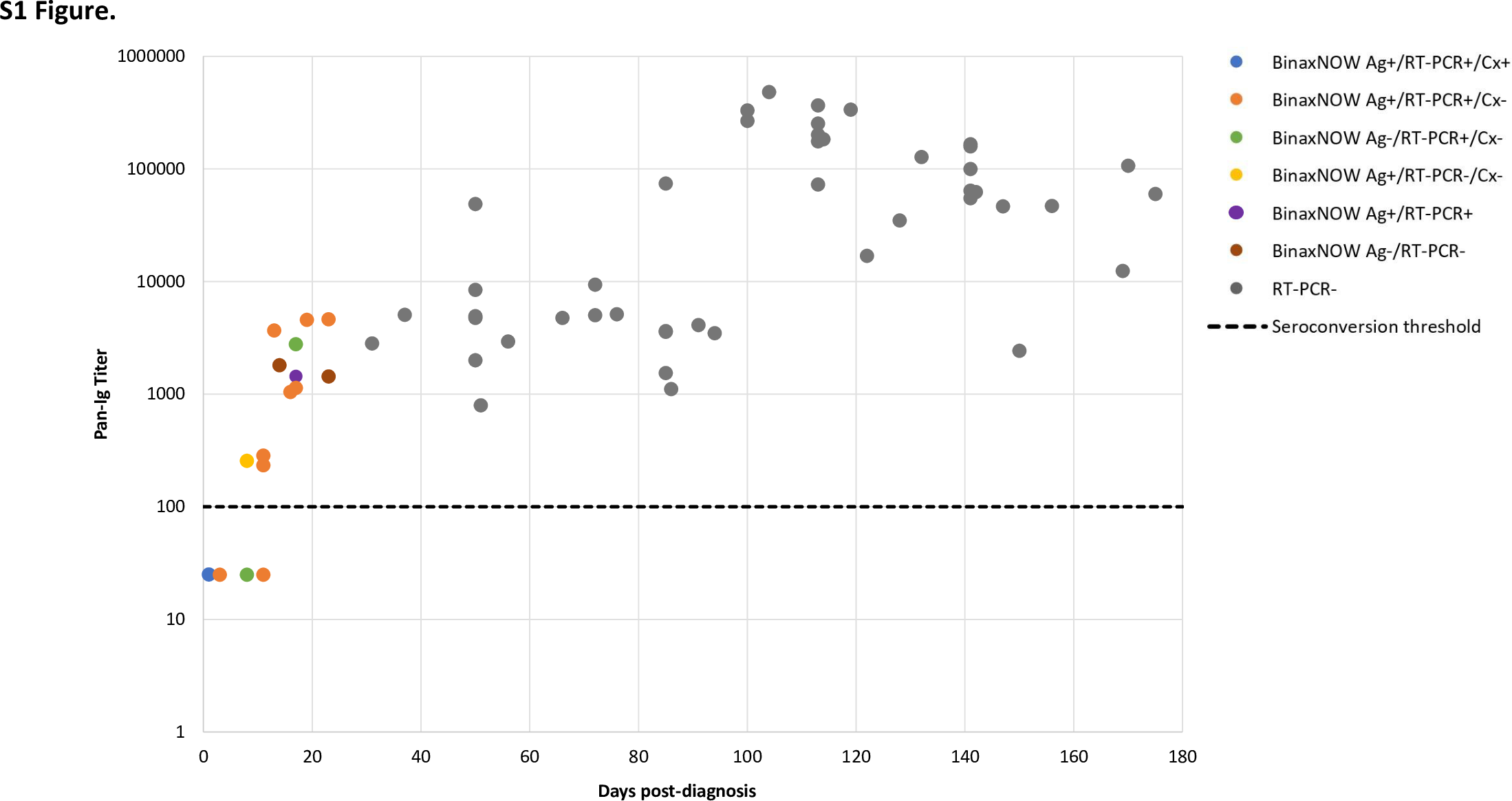

Supplement: S1 Fig — Abbreviations: BinaxNOW = BinaxNOW™ COVID-19 Ag Card; RT-PCR = real-time reverse transcription polymerase chain reaction; Cx = viral culture. Blood samples were obtained at several timepoints between 1–150 days post-diagnosis (time period after each participant’s first positive SARS-CoV-2 test result). Due to challenges phlebotomizing some participants, serum titers were not generated for each participant for each visit. Participants with serum antibody titers below the seroconversion threshold (defined as a signal threshold >1 at the 1:100 dilution for any isotype) were assigned a value of 25 for graphical representation only. The y-axis is plotted in logarithmic scale. (TIF) [file pone.0275718.s002.tif]

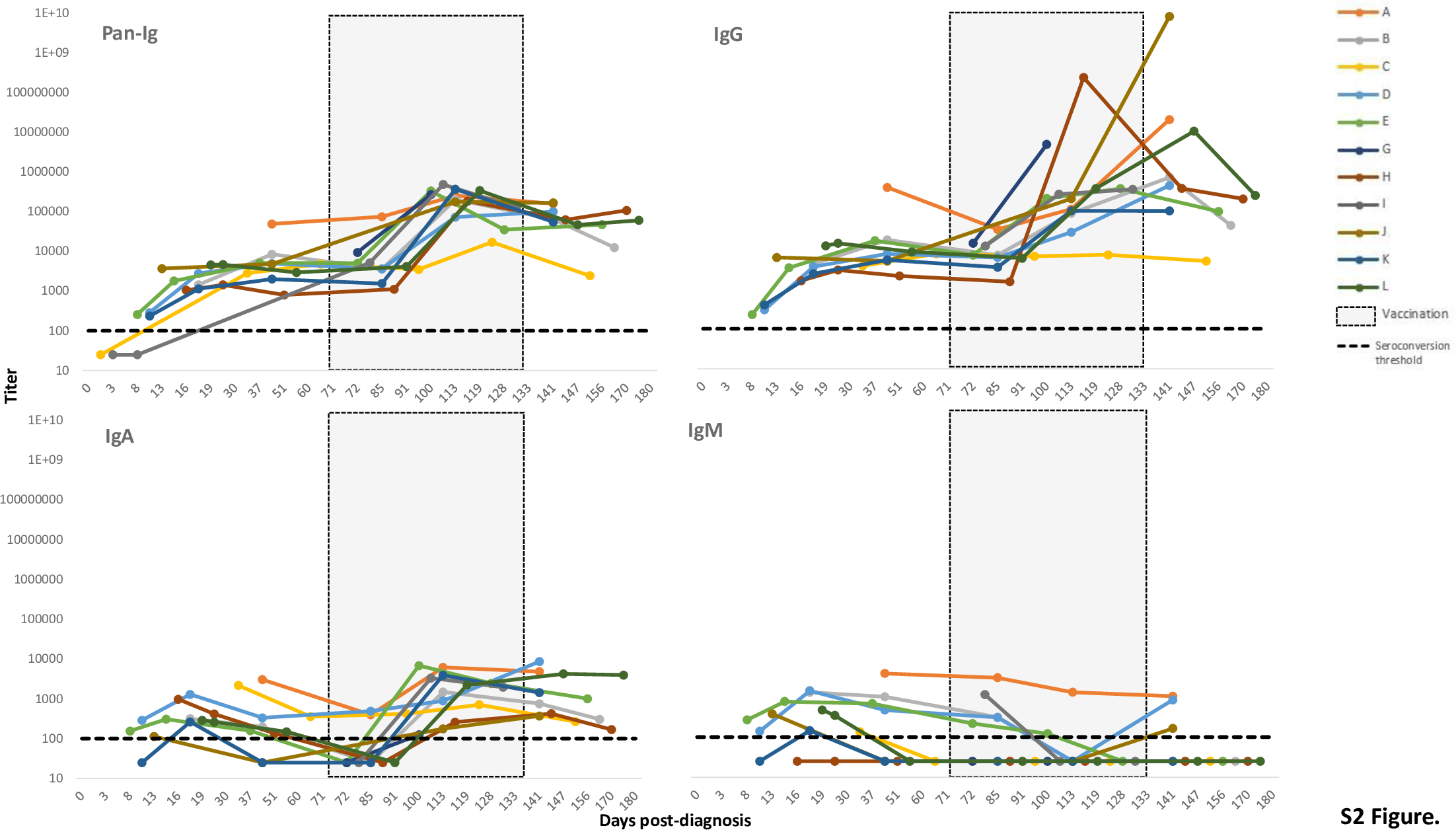

Supplement: S2 Fig — Colored lines represent individual participants. Blood samples were obtained at several timepoints between 1–150 days post-diagnosis (time period after each participant’s first positive SARS-CoV-2 test result). Due to challenges phlebotomizing some patients, serum titers were not generated for each participant for each visit. Pfizer-BioNTech COVID-19 vaccines were administered at the facility between 71–105 days post-diagnosis; participant C declined vaccination. Participants with serum antibody titers below the seroconversion threshold (defined as a signal threshold >1 at the 1:100 dilution for any isotype) were assigned a value of 25 for graphical representation only. The y-axis is plotted in logarithmic scale. (TIF) [file pone.0275718.s003.tif]
