# Supplementary material for: Longitudinal serologic and viral testing post–SARS-CoV-2 infection and post-receipt of mRNA COVID-19 vaccine in a nursing home cohort—Georgia, October 2020‒April 2021
Source: PLoS One. 2022 Oct 27;17(10):e0275718. doi: 10.1371/journal.pone.0275718 (PMC9612440; doi:10.1371/journal.pone.0275718)
Supplement: S3 Fig — (DOCX) [file pone.0275718.s004.docx]

**Ct value**

**Titer**


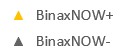

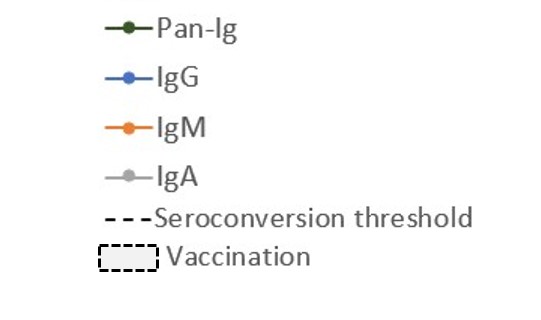

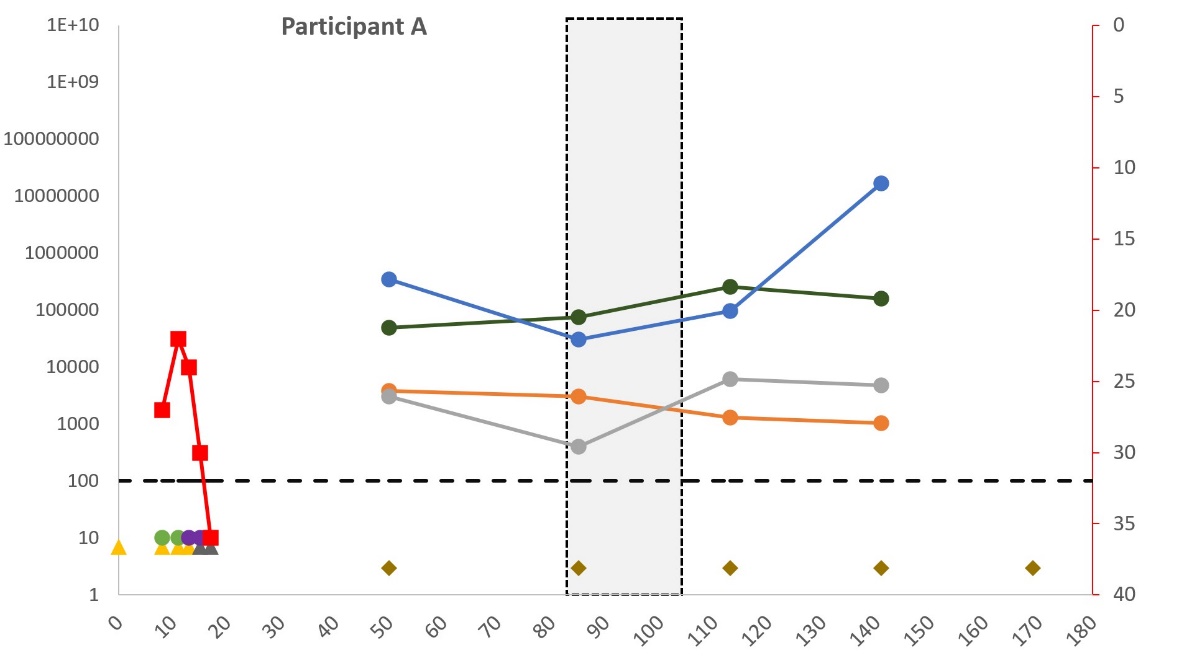


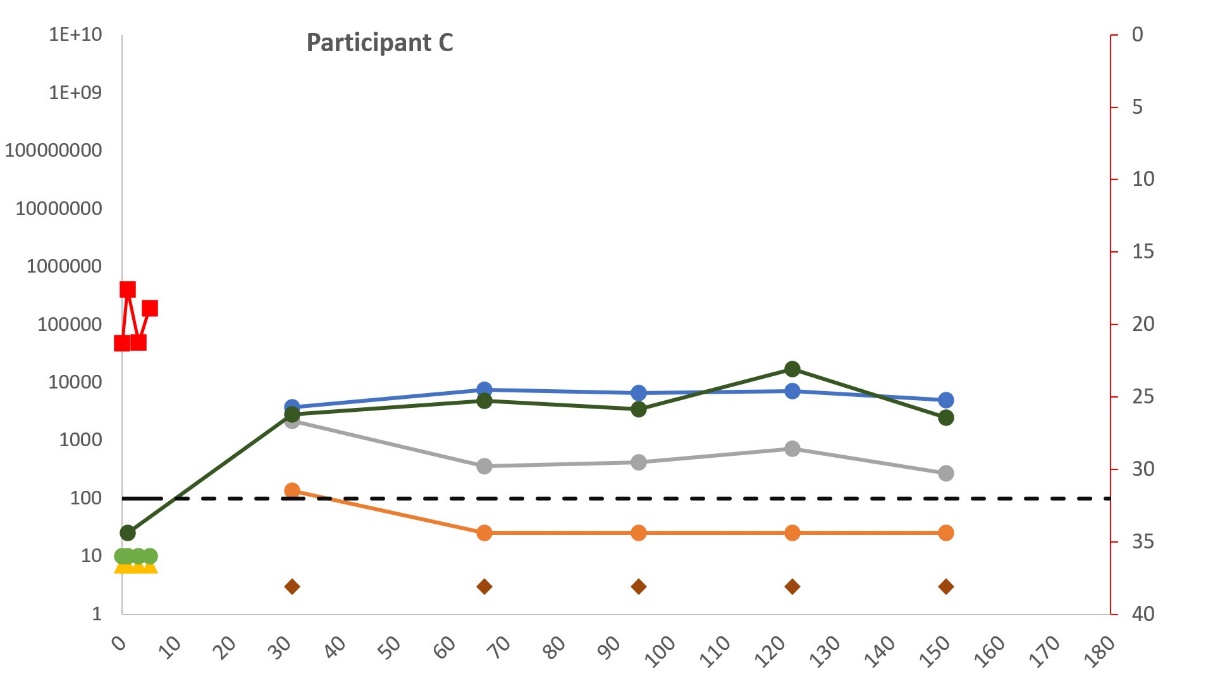


**Days post-diagnosis**

**Days post-diagnosis**

**Days post-diagnosis**

**Titer**

**Ct value**


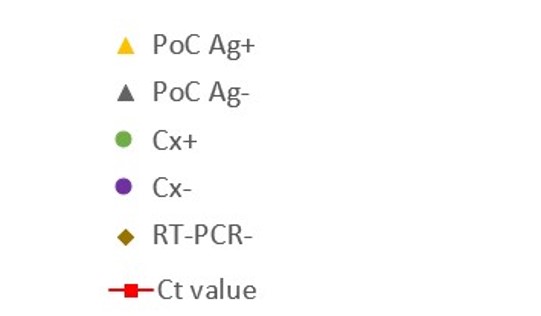


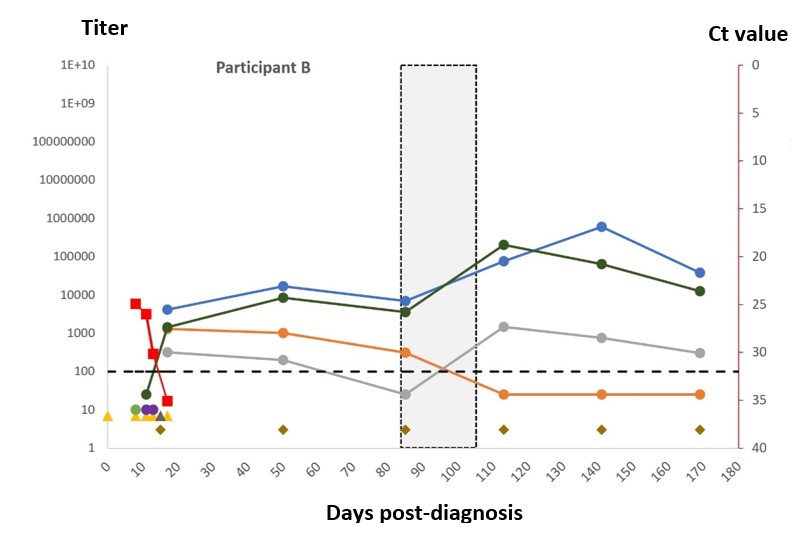


**Ct value**

**Titer**


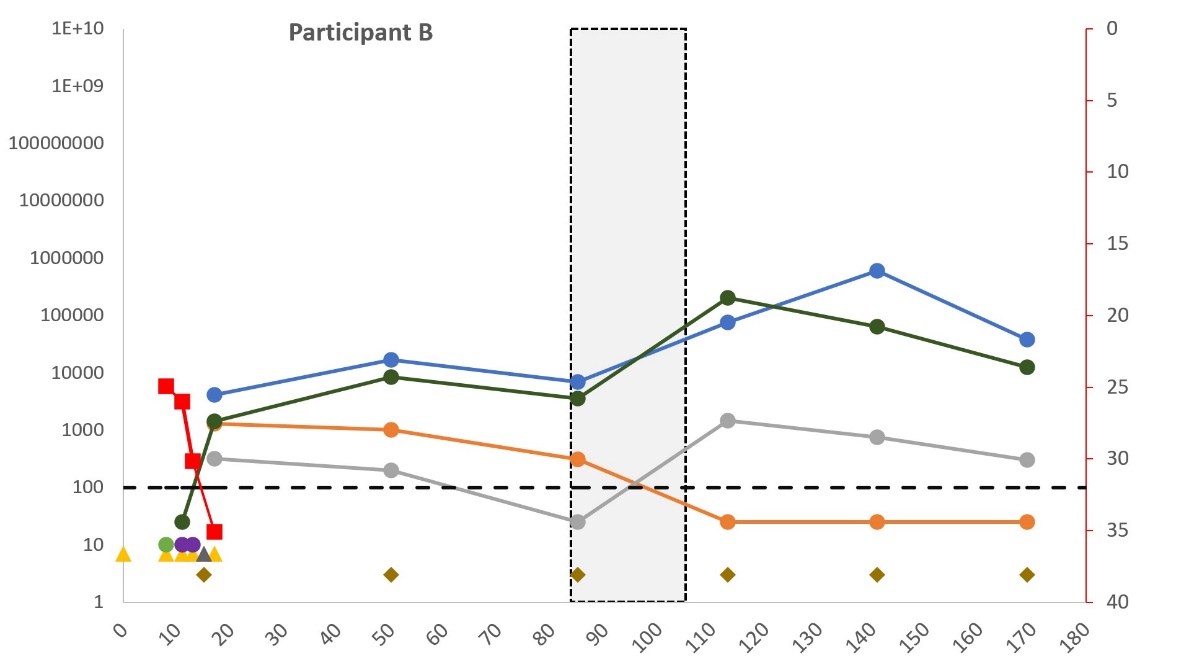


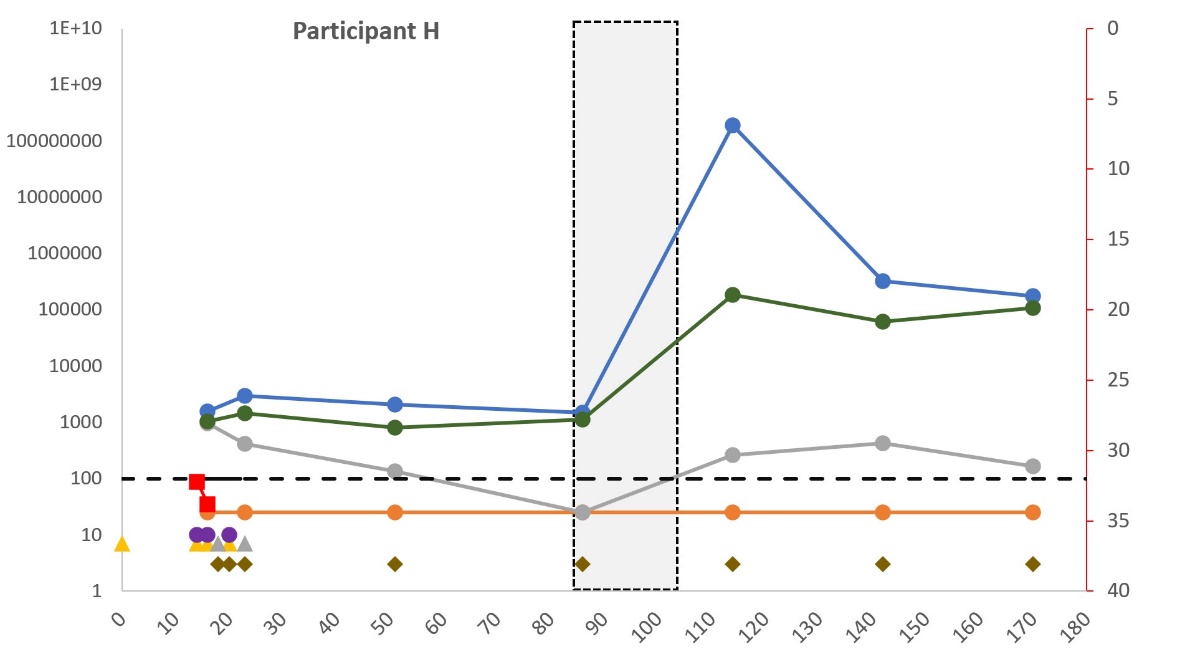

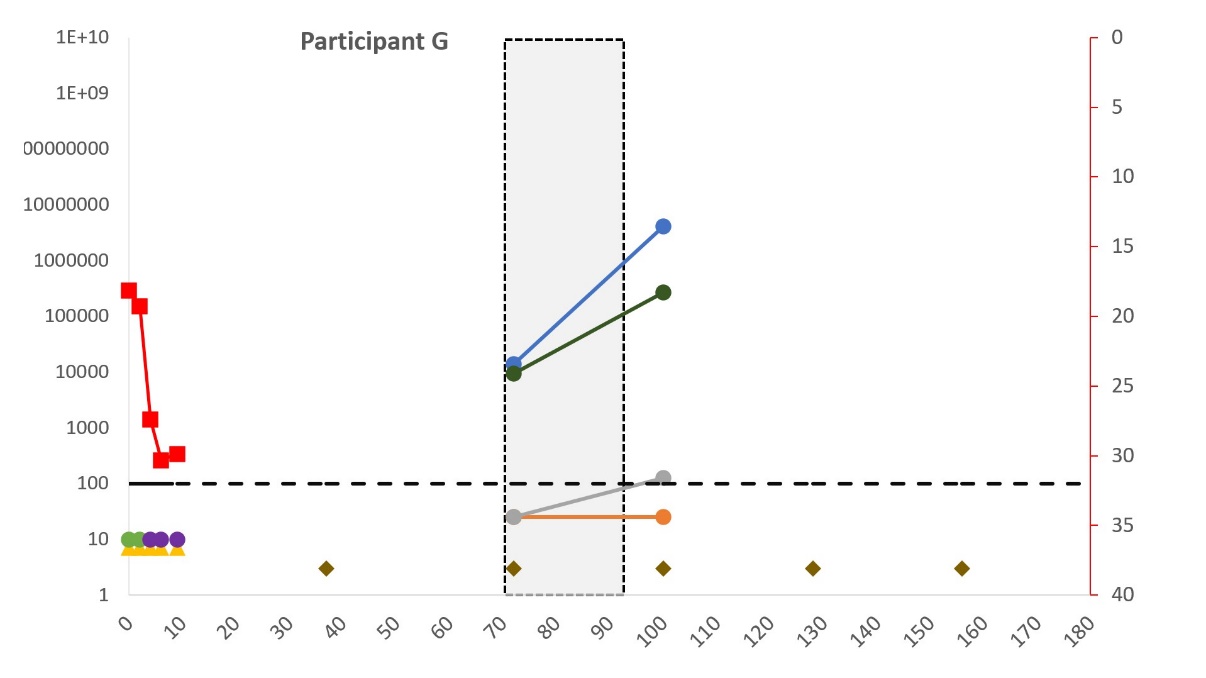

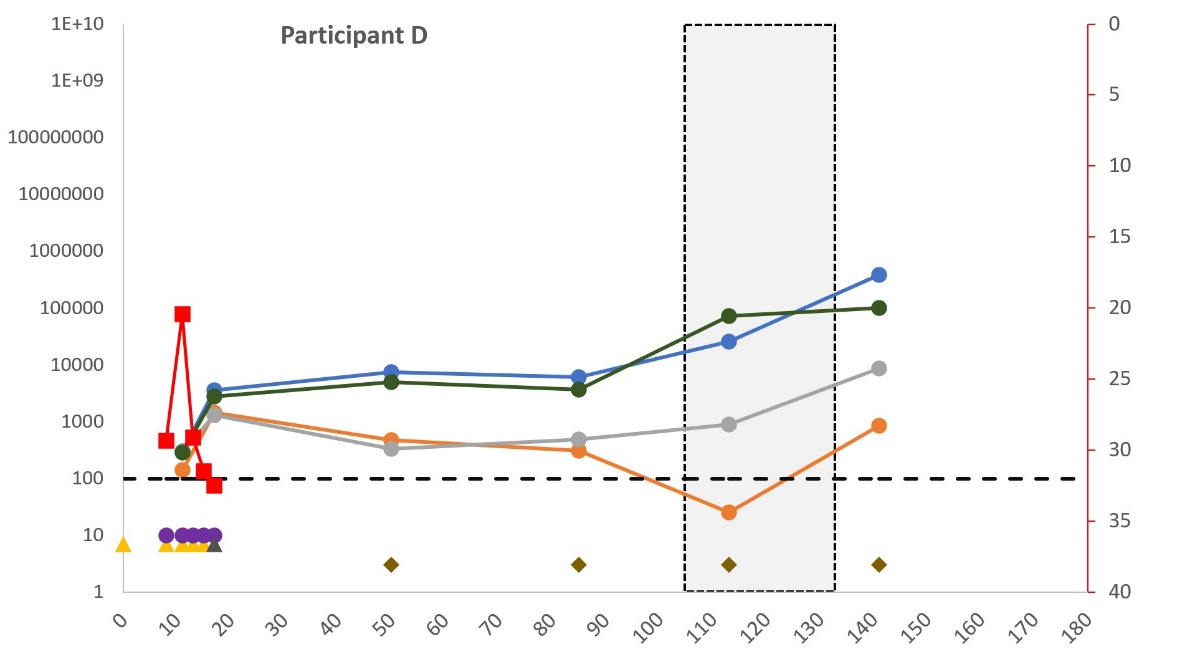

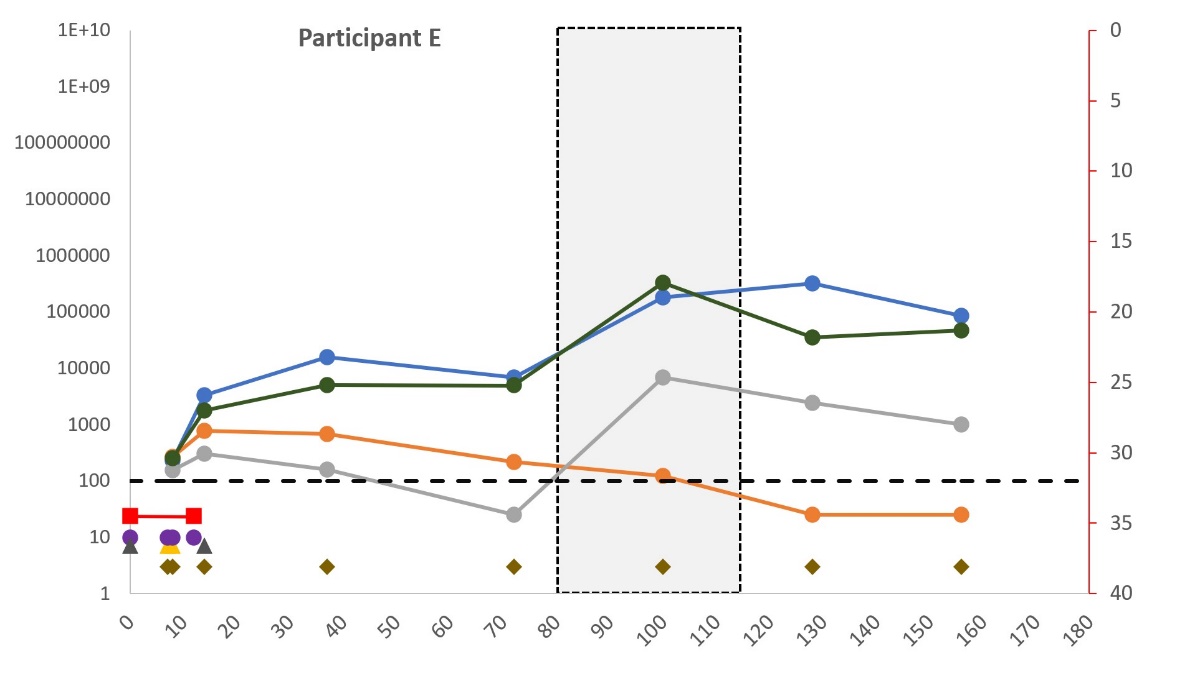


**Ct value**

**Ct value**

**Ct value**

**Ct value**

**Titer**

**Titer**

**Titer**

**Titer**

**Days post-diagnosis**

**Days post-diagnosis**

**Days post-diagnosis**

**Days post-diagnosis**


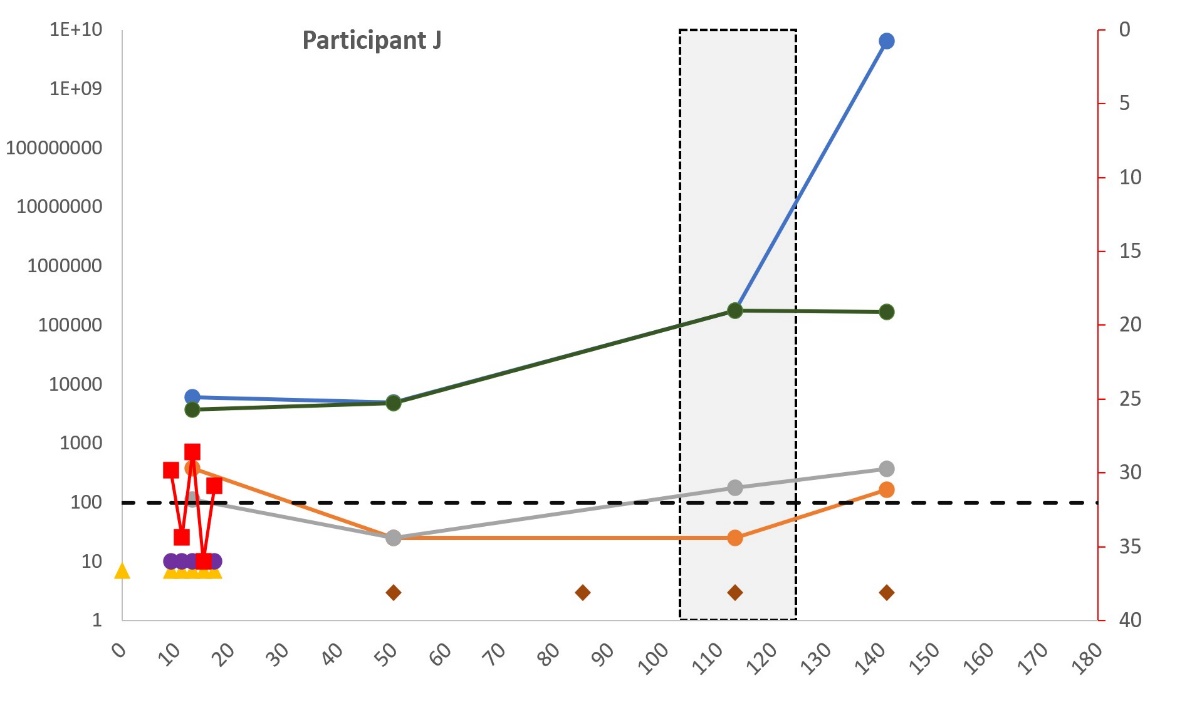

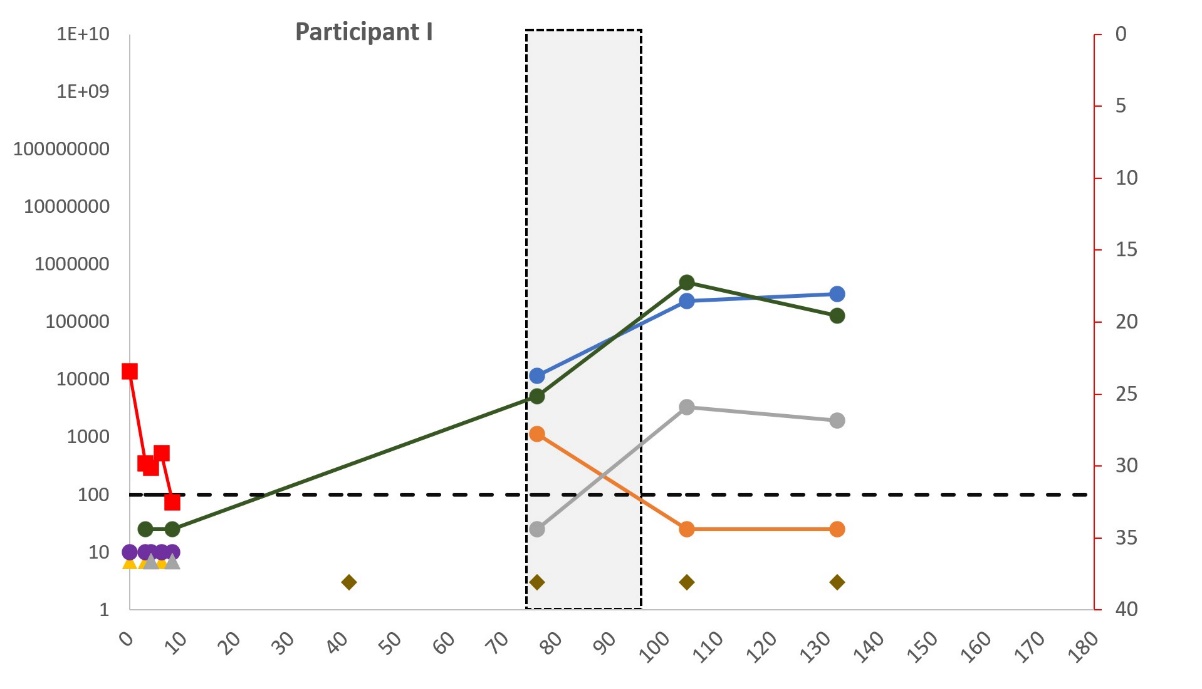


**Ct value**

**Ct value**

**Titer**

**Titer**

**Days post-diagnosis**

**Days post-diagnosis**

**Ct value**

**Titer**

**Ct value**

**Titer**


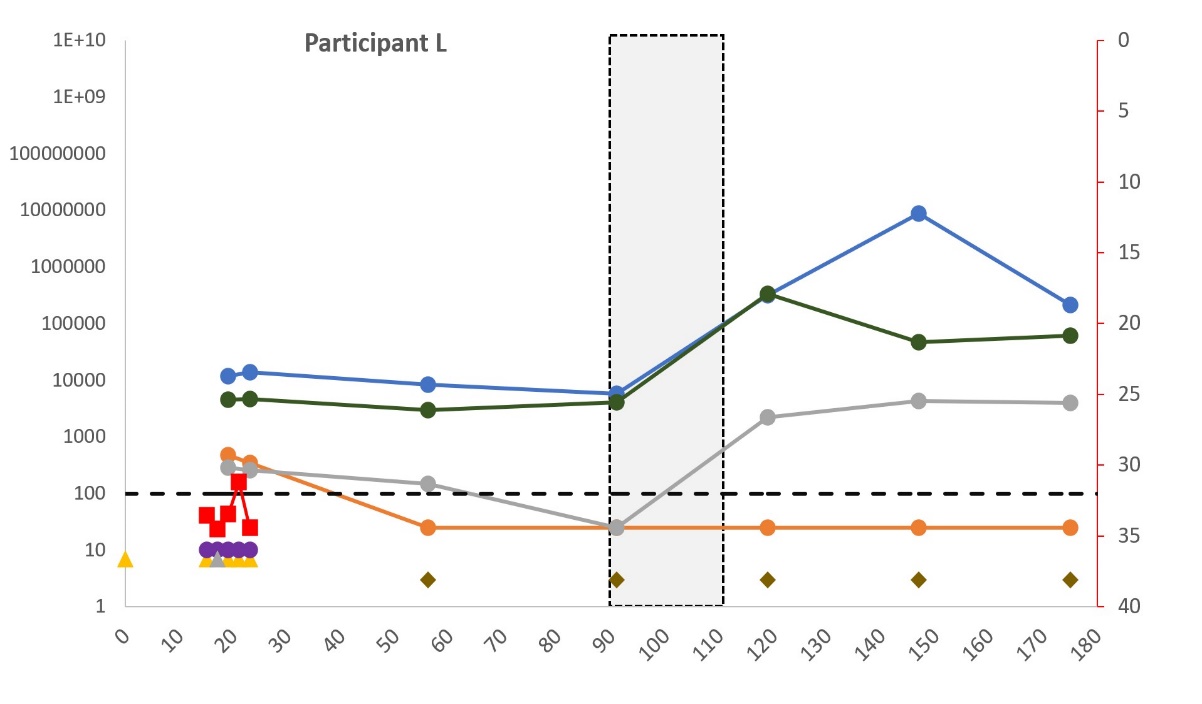

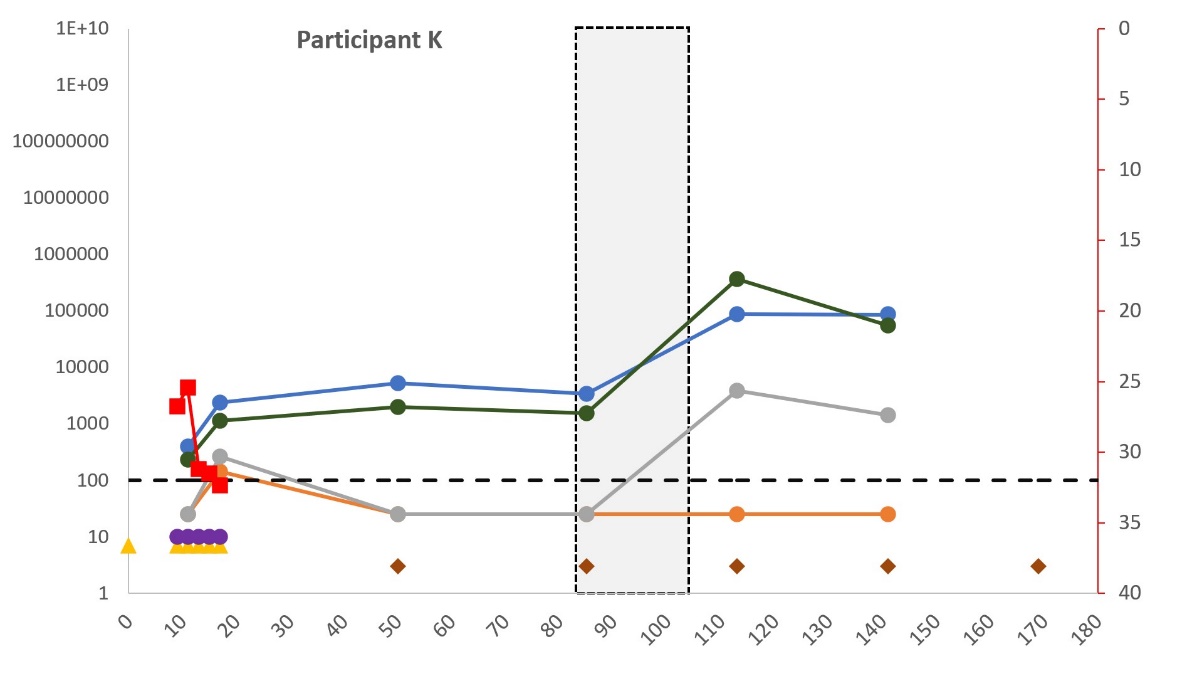


**Days post-diagnosis**

**Days post-diagnosis**
